# A prospective, randomised, controlled clinical study on the assessment of tolerability and of clinical efficacy of Merional (hMG-IBSA) administered subcutaneously versus Merional administered intramuscularly in women undergoing multifollicular ovarian stimulation in an ART programme (IVF)

**DOI:** 10.1186/1477-7827-5-45

**Published:** 2007-12-04

**Authors:** Carlo Alviggi, Alberto Revelli, Paola Anserini, Antonio Ranieri, Luigi Fedele, Ida Strina, Marco Massobrio, Nicola Ragni, Giuseppe De Placido

**Affiliations:** 1 Department of Obstetrica/Gynecological Sciences and Reproductive Medicine, Federico II University, Napoli, Italy; 2 Reproductive Medicine and IVF Unit, Department of Obstetrical and Gynecological Sciences, University of Torino, S. Anna Hospital, Torino, Italy; 3 Reproductive Medicine Unit, S. Martino Hospital, Genova, Italy; 4 Reproductive Medicine Unit, S. Paolo Hospital, Milano, Italy

## Abstract

**Background:**

Multifollicular ovarian stimulation (MOS) is widely used in IVF and the compliance to treatment is deeply influenced by the tolerability of the medication(s) used and by the ease of self-administration. This prospective, controlled, randomised, parallel group open label, multicenter, phase III, equivalence study has been aimed to compare the clinical effectiveness (in terms of oocytes obtained) and tolerability of subcutaneous (s.c.) self-administered versus classical intramuscular (i.m.) injections of Merional, a new highly-purified hMG preparation.

**Methods:**

A total of 168 normogonadotropic women undergoing IVF were enrolled. Among them, 160 achieved pituitary suppression with a GnRH-agonist long protocol and were randomised to MOS treatment with Merional s.c. or i.m. They started MOS with a standard hMG dose between 150–300 IU, depending upon patient's age, and underwent a standard IVF procedure.

**Results:**

No statistically significant difference in the mean number of collected oocytes (primary endpoint) was observed between the two study subgroups (7.46, SD 4.24 vs. 7.86, SD 4.28 in the s.c. and i.m. subgroups, respectively). As concerns the secondary outcomes, both the pregnancy and the clinical pregnancy rates were comparable between subgroups. The incidence of adverse events was similar in the two groups (2.4% vs. 3.7%, respectively). Pain at injection site was reported only the i.m. group (13.9% of patients).

**Conclusion:**

Merional may be used by s.c. injections in IVF with an effectiveness in terms of retrieved oocytes that is equivalent to the one obtained with i.m administration and with a better local tolerability. With the limitations due to the sample size af this study, s.c. and i.m. administration routes seem to have the same overall safety.

## Background

Multifollicular ovarian stimulation (MOS) with gonadotrophins is an integral part of various assisted reproductive technologies (ART), including standard *in-vitro *fertilization (IVF) and intracytoplasmic sperm injection (ICSI). The hormonal control of multiple follicular growth is crucial in determining the quality and yield of oocytes and consequently the availability of embryos to transfer in utero, and has a relevant effect on the treatment outcome [1].

Human Menopausal Gonadotrophin (hMG) has been in use for over 30 years in women with WHO group I or II ovulatory disorders. This medication currently represents a widespread, safe and effective treatment choice for MOS in ART [2]. Nevertheless, hMG preparations are extracted from the urine of post-menopausal women, and small peptidic contaminants are usually detectable in the commercially available preparations [3,4]. As a consequence of these technical limits, the s.c. employment of extractive hMG, although well acceptable by patients wishing to self-administer the drug, is limited, and i.m. administration is suggested [5].

Recently recombinant gonadotrophins, which are virtually free from aspecific contaminants, have been made available and have been introduced on the market [6,7]. These drugs can be self-administrated by s.c. injection [8] and although on the average more expensive than hMG [9], they appear to be associated with a better compliance. Given that the current literature is still insufficient to definitively establish which gonadotrophin preparation (urinary or recombinant) is more effective in IVF and the available data are contradictory [10-13], attention has been recently turned on costs, safety and tolerability of available formulations.

Merional^® ^(hMG) is a newly purified hMG preparation containing 75 IU of follicle stimulating hormone (FSH) activity and 75 IU of LH activity. It is produced using a patented purification method and is registered and commercially available in 15 countries worldwide. Merional^® ^is characterized by an high degree of purification leading to the following final specific activity of the individual hormone components: LH > 1,000 IU/mg, FSH > 3,000 IU/mg, hCG > 6,000 IU/mg. This purity level may theorically allow s.c. self-administration, but its clinical effectiveness when administered s.c. to IVF patients has not yet been clarified.

This prospective, controlled, randomised, parallel group open label, multicenter, phase III, equivalence study has been aimed to compare the clinical effectiveness, tolerability (both local and systemic) and safety of Merional^® ^s.c. administration versus i.m. injection in normogonadotrophic patients undergoing MOS in an IVF program.

## Methods

### Overall study design and patients' selection

This equivalence study was carried out according to a prospective, controlled, randomised, parallel group open label, multicentre design. The ethics committe of each Department participating in the study gave its written approval to the study design. A total number of 168 normogonadotropic women were scheduled to be included in the study in 4 investigational Centres, that used homogeneous procedures to accomplish IVF cycles throughout the whole time period of investigation. The number of patients recruited by each Centre was 42, 38, 51, and 37, respectively. Following written informed consent, patients with normal ovulatory cycles, aged 20–40 years, with a Body Mass Index (BMI) between 20 and 28 kg/m^2^, were enrolled and submitted to IVF/ICSI therapy with husband's semen. The following exclusion criteria were adopted: a) basal FSH levels ≥ 9 IU/L and/or documented ovarian failure; b) ascertained or presumptive hypersensitivity to Merional^® ^components; c) presence of an ovarian cyst(s) or of ovarian enlargement not due to polycystic ovarian syndrome; d) patients undergoing oocyte donation; e) presence of an abnormal uterine bleeding of unknown origin; f) documented poor ovarian responsiveness (requirement of at least 300 IU/d of FSH or hMG) in previous IVF cycles; g) hormonal treatments (e.g. corticosteroids or oral contraceptives) in the 30 days preceding the beginning of the study; h) presence of a documented thyroid or adrenal dysfunction or of other endocrinological diseases; i) presence of systemic diseases (e.g. malignant tumors, severe renal and/or hepatic dysfunctions, diabetes mellitus, thrombophlebitis, cardiac diseases, epilepsy, etc.); l) presence of any contraindication toward pregnancy; m) presence of any anatomical abnormality of the reproductive apparatus (e.g. uterine myoma, etc.).

### Determination of sample size

The number of retrieved oocytes at oocyte pick-up (OPU) was retained as the primary end-point and equivalence parameter, and an equal mean number of retrieved oocytes in the two treatment arms was the null hypothesis.

According to this equivalence experimental design and choosing α = 0.05 and β = 0.20, a minimum number of 80 patients per treatment arm was calculated to be appropriate.

### Removal of patients from the study protocol

Patients could be removed from the study for any of the following reasons: a) no ovarian response to medication or excessive risk of ovarian hyperstimulation syndrome (OHSS); b) appearance of adverse events or of a concomitant illness; c) violation of the protocol (e.g. contemporaneous use of other medications not compatible with the inclusion criteria); d) patient's will to quit the protocol; e) uncompleted (or complete loss of) follow up.

The investigators had to specify the cause of patient's removal in the 'Study Drop-Out Form' in the 'Case Record Form'.

### Ovarian stimulation

Patients were randomised using a computer-assisted 1:1 randomization to undergo MOS with either Merional^® ^administered subcutaneously (s.c.) or by intramuscular (i.m.) injections. Since treatment subgroups were defined according to administration route, no blinding procedure could be adopted toward both patients and doctors monitoring MOS. Anyway, embryologists were "blind" as they did not know to which treatment arm were the patients assigned.

All patients received a standard GnRH-agonist long protocol using Triptorelin (Decapeptyl^® ^0.1, IPSEN, Italy) at the daily dose of 0.1 mg s.c. starting on the 21^st ^day of the cycle preceding IVF treatment. The achievement of pituitary desensitisation was confirmed by transvaginal ultrasound (no evidence of ovarian activity, endometrial thickness < 5 mm) and circulating estradiol assessment (< 50 pg/ml). Patients showing pituitary down-regulation started Merional^® ^treatment, whereas patients with ovarian cysts at ultrasound and/or endometrial thickness ≥ 5 mm and/or E_2 _levels > 50 pg/mL went on with GnRH-agonist for 5 further days and were checked again to assess pituitary suppression; only in case they achieved it, the ovarian stimulation with Merional^® ^was began. Overall, 160 patients out of the 168 initially chosen could start MOS. Merional^® ^starting dose was scheduled according to the patient's age: it was 150 IU/d in patients < 35 years, 225 IU between 35 and 38 years, 300 IU between 38 and 40 years. Although not as accurate as antral follicle count (AFC) or basal FSH level, age was used to chose the starting FSH dose because of its lack of subjectivity (vs. AFC) and lack of difference among assay methods (vs. basal FSH) that allowed the four recruiting Centers to be very homogeneous. After the sixth stimulation day, the hMG dose was eventually adjusted according to the ovarian response assessed by serum E_2 _and US measurement of follicular number and size; dose corrections were scheduled in advance as the daily dose could be increased or decreased by a maximum of 150 IU of hMG. Transvaginal US and E_2 _serum levels measurement were performed on the first day of stimulation and after 7 days, as well as every second day thereafter. Merional^® ^administration continued until the criteria for triggering the final follicular maturation (one follicle ≥ 18 mm diameter plus at least two other follicles ≥ 16 mm, with appropriate estradiol levels) were met; in case of a poor response, ovarian stimulation was anyway stopped after 18 days. Ten thousand IU of hCG (Gonasi, AMSA, Italy) were given i.m. 34–36 hrs prior to transvaginal OPU. After recovering the oocytes, fertilization was achieved by either standard IVF or ICSI techniques and embryo transfer was performed after 48–72 hrs of in vitro culture using a soft catheter. Transvaginal progesterone (400 mg) was administered daily for 14 days and a urinary pregnancy test was then performed; in case of a positive test, the number of gestational sacs and the presence of embryonic heart beat were assessed by transvaginal US after further 3 weeks.

### Effectiveness and safety

In order to evaluate the treatment effectiveness, the number of retrieved oocytes was chosen as the primary endpoint, and the power calculation was made according to it. In addition, the following secondary end-points were considered: stimulation length (days), cumulative hMG dose, E_2 _area under the curve (AUC_0-t _calculated according to a trapezoidal rule and adjusted with respect to baseline) and peak concentration (C_MAX_), fertilization rate, pregnancy rate (positive urinary hCG)/cycle) and clinical pregnancy rate (US-assessed foetal heartbeat)/cycle.

The safety of Merional^® ^was assessed using blood tests and by giving to the patients a self-evaluation well-being questionnaire. The time of onset, severity and duration of any adverse reaction was recorded, and any action and/or pharmacological treatment adopted to face it was registered. The risk of OHSS was considered so high to justify cycle cancellation when more that 20 follicles developed above 12 mm diameter and/or serum estradiol exceeded 3500 pg/ml. As to tolerability, any red area surrounding the most recent injection site was recorded, as well as the eventual presence of pain, itching, or tenderness at the injection site.

### Hormonal assays

The enzyme-linked fluorescent assay (ELFA) technique (Vidas Oestradiol II, BioMérieux, France) was used for measuring serum E_2 _concentrations. This commercial assay has the following characteristics: detection limit (the lowest concentration detectable as different from zero with a 95% probability) 0.03 pg/mL, intra- and inter-assay variation coefficients <7%.

### Statistical analysis

Statistical analyses to assess effectiveness and safety were carried out on the population exposed to Merional^®^, thus excluding those patients who were enrolled in the study but did not achieve pituitary suppression and consequently did not receive hMG.

The absence of significant differences in the baseline characteristics between the two subgroups was assessed by the Student *t *and the χ^2 ^tests.

In order to assess differences between the two study subgroups, variables showing a normal distribution of the data at Lilliefors test were tested by unpaired Student-*t*-test, while in presence of a non-normal distribution, the non parametric Mann-Whitney *U*-test was used. Differences having a binomial distribution (e.g. pregnancy rate, foetal heart beat rate) were tested by χ^2 ^test. The Schuirmann's test was used to test equivalence in the primary outcome as well as in the secondary outcome variables. The patients' subjective tolerance judgement and the presence of a high OHSS risk in the two subgroups were compared according to Mantel-Haenszel χ^2 ^test for trends. Any difference between the two treatment subgroups were declared to be significant in presence of a two-tailed *p *value < 0.05.

Data management was performed using the Microsoft SQL Server^® ^version 2000. Statistical calculations were performed with SAS^® ^8.2 installed on a Pentium 4. The statistical package EquivTest was used for equivalence calculations.

## Results

**Figure 1:**
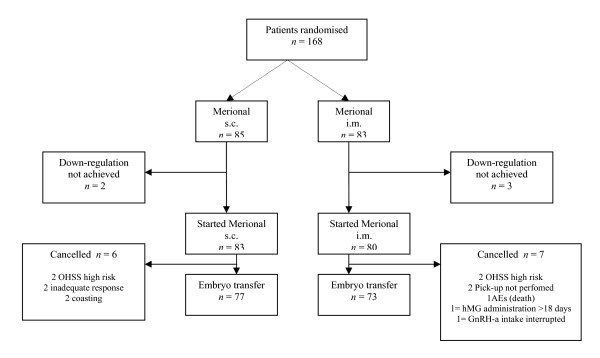
Schematic representation of the distribution of the 168 enrolled patients throughout the study.

A total number of 168 patients were enrolled in the study on the basis of the inclusion criteria (Fig. 1); 85 of them were randomly allocated in the s.c. administration group, while 83 were assigned to the i.m. administration group. The treatment subgroups were not significantly different in terms of any of the baseline characteristics analyzed, included the semen quality and the proportion of IVF and ICSI procedures (Table 1).

**Table 1:** Baseline characteristics of the patients included in the study.

	**Merional s.c.**	**Merional i.m.**	P value
N. of patients randomised	85	83	
Age (years)*	34.6 ± 4.9	34.4 ± 5.2	NS
Diagnosis of male factor (%)	48	51	NS
Diagnosis of unexplained infertility (%)	18	17	NS
Diagnosis of tubal factor (%)	24	25	NS
Endometriosis (%)	10	7	NS
Proportion of ICSI (%)	53	54	NS

* Values expressed as mean ± SD.

Two and 3 patients in the mentioned subgroups, respectively, did not achieve the pituitary down-regulation and were consequently removed from the protocol. In the two subgroups, 83 patients in the s.c. and 80 in the i.m. arm, respectively, started treatment with Merional^®^. Among them, 77 (92.8%) and 73 (91.2%), respectively, completed the MOS up to oocyte pick-up (OPU). The reasons for dropping out during MOS are shown in Figure 1. The starting dose of Merional^® ^was adjusted (increased) in one patient of the i.m. subgroup due to inadequate ovarian response, and in three patients of the s.c. subgroup due to patient's mistake in self-administration. Dose reduction due to excessive ovarian response was necessary in 8 patients of the i.m. group and 13 patients of the s.c. group. The reported death of one patient after starting stimulation was not related to the treatment as it was due to a car accident.

The analysis of the primary end-point (number of retrieved oocytes) made by the Schuirmann test showed therapeutic equivalence between s.c. and i.m. Merional^® ^in terms of oocytes retrieved at OPU. No statistically significant difference was observed for most secondary end-points including the pregnancy rate and the clinical pregnancy rate (Table 2). Only the total hMG dose was significantly lower in the s.c. than in the i.m. subgroup (2168.0 ± 729.55 vs. 2595.1 ± 951.2 IU, respectively; p < 0.01), and the stimulation length was shorter in the s.c. than in the i.m. subgroup (13.8 ± 1.7 vs.14.6 ± 2.1 days, respectively; p < 0.05) (Table 2).

**Table 2:** Outcome of ovarian stimulation and IVF in patients who received Merional by s.c.vs. i.m. injections.

	**Merional s.c.**	**Merional i.m.**	P value
N. of patients starting MOS	83	80	
Duration of MOS (days)*	13.8 ± 1.7	14.6 ± 2.1	0.02
hMG total dose (IU)*	2168.6 ± 729.55	2595.1 ± 951.2	0.002
AUC_0-t _of E_2 _(log-transformed pmol/L*days)*	8.7 ± 0.6	8.8 ± 0.5	NS
C_MAX _of E_2 _(pmol/L) *	2215.5 ± 1288.4	2416.2 ± 1258.5	NS
Completed COS cycles (OPUs)	77	73	
N. of oocytes retrieved/OPU*	8.0 ± 3.8	8.6 ± 3.7	NS
N. of fertilized oocytes/OPU*	3.6 ± 2.7	4.0 ± 3.0	NS
N. of embryo transfers (ETs)	77	73	
hCG positive tests	32	25	
Pregnancy rate/started cycle (%) ^a^	38.5	31.2	NS
Pregnancy rate/ET (%)^a^	41.5	34.2	NS
Clinical pregnancies	21	19	
Clinical pregnancy rate/started cycle (%) ^b^	25.3	23.7	NS
Clinical pregnancy rate/ET (%)^b^	27.3	26.0	NS

* Values expressed as mean ± SD.

^a ^Women who had positive-urine pregnancy test performed 14 days after embryo transfer.

^b ^Women who had US evidence of foetal heartbeat

The recorded incidence of adverse effects was overall very low and similar in the two subgroups (2.4% vs. 3.7% of patients in the s.c. and i.m. subgroups, respectively); these adverse effects were headache, or aspecific abdominal pain, both not clearly related to the drug's intake. Blood chemistry tests during MOS showed no changes in comparison to baseline values in both subgroups (not shown). Excessive OHSS risk was found in 2 out of 83 (2.4%) patients of the s.c. subgroup and in 2 out of 80 (2.5%) patients in the i.m. subgroup. Pain at injection site was recorded in 13.9% of patients in the i.m. subgroup, and in none in the s.c. subgroup.

## Discussion

Although IVF may be performed retrieving an oocyte from the single follicle developing during a spontaneous ovulatory cycle [14] or collecting immature oocytes in the middle follicular phase and maturing them in vitro [15,16], multifollicular ovarian stimulation (MOS) with gonadotropins is by far the most widely used method to obtain fertilizable oocytes in IVF treatment cycles. The possibility of fertilizing several eggs that have matured in follicles growing during controlled ovarian stimulation is still considered an important advantage in comparison to the before mentioned alternatives, that are used almost only in very selected subsets of patients, such as women with the polycystic ovary syndrome [17].

Several protocols for gonadotropin administration in IVF have been studied and proposed in the last twenty-five years, and relevant efforts have been made to introduce on the market new products coupling effectiveness, safety, and subjective tolerability. A recent study about the compliance of patients to MOS has demonstrated that the route of administration of gonadotropins deeply affects the patient's tolerance to the treatment, and that the subcutaneous (s.c.) route is the one with the best impact on the patient's quality of life, and tends to be preferred to the intramuscular (i.m.) route [18]. Some bioequivalence studies have also shown that both FSH [19] and hMG [20] have the same biological and pharmacokinetic properties when injected i.m. or s.c., and show the same effectiveness in stimulating follicular recruitment and growth.

The present study was designed to assess the effectiveness of the s.c. administration of Merional^®^, a new highly-purified form of hMG, in inducing MOS in IVF patients, and to compare it with the same drug given i.m at comparable doses. The possibility to obtain the same number of oocytes available for fertilization using a highly purified hMG given in an easy and very tolerable way is intriguing also in view of prescribing the drug for self-administration.

The randomisation procedure achieved two subgroups (s.c. vs. i.m. Merional^®^) that resulted to be well balanced as far as all relevant patients' baseline characteristics are concerned. On the whole, the study population was adequate in terms of homogeneity and size to support an informative comparison toward the primary end-points of effectiveness (number of oocytes) and safety (incidence of adverse effects). Although it was not possible to organize a fully "blind" study (patients obviously knew the way of administration, and doctors following MOS also knew because patients in the i.m. subgroup sometimes asked to be helped for i.m. injections), both doctors performing OPU (in most cases) and embryologists (always) were "blind" as they did not know to which subgroup each patient belonged. As a consequence, the number of retrieved oocytes (primary end-point) as well as all IVF procedures in the laboratory may be considered as "blind study" findings, not affected by the patient's or the doctor's will.

The main observation emerging from this prospective randomised trial is the bioequivalence of s.c and i.m. Merional^® ^administration in terms of oocyte production during MOS and recovery at OPU. In fact, patients submitted to MOS using s.c Merional^® ^had a comparable number of oocytes retrieved at OPU than women receiving the same preparation by the classical i.m. route. We are aware that an equal number of retrieved oocytes does not necessarily mean to have the same chance of obtaining fertilized oocytes, transferable embryos, and finally a pregnancy; oocyte quality, in fact, is the major determinant of IVF outcome and its relative importance is by far higher than oocyte number [21]. The present trial does not show a full therapeutic equivalence between s.c. and i.m. Merional^® ^because it was powered according to the primary end-point "number of retrieved oocytes" and it is underpowered to show equivalence between the two routes of Merional^® ^administration in terms of IVF treatment success. We cannot therefore state that s.c. and i.m. hMG administration routes are equivalent in terms of overall effectiveness on IVF cycle, but still we can say that we got the same number of oocytes in the two subgroups and we did not observe a trend toward better IVF results in either of the two subgroups.

In the present study, patients treated with s.c. administration showed a significantly shorter stimulation and they also received a significantly lower total Merional^® ^dose. Interestingly, a similar observation was reported by Voortman et al. [19], that compared i.m vs. s.c. recombinant FSH and reported a better ovarian response with the s.c. administration route despite an absolute bioequivalence in pharmacokinetic. Similarly, pharmacokinetic studies evaluating Merional^® ^s.c. and i.m.administration routes in healthy female volunteers showed comparable pharmacokinetic profiles (unpublished IBSA report on file), but still Merional^® ^intake was significantly lower using s.c. administration (-16.4% on average), with a consequent reduction in the economical cost per cycle.

As for safety, the incidence of adverse effects was very limited and not significantly different in the two study subgroups. Laboratory tests did not provide evidence of treatment-related changes in either subgroup, and neither the self-evaluation well-being assessment, nor the clinical observation of the patients' well-being accomplished by doctors monitoring MOS showed any difference according to the drug administration route. The incidence of OHSS was low in both subgroups and also the need to cancel the stimulation cycle because of excessive OHSS risk was very limited.

As for subjective tolerability, pain at injection site was signalled by 13.9% of patients in the i.m. subgroup, and by nobody in the s.c. subgroup, suggesting some advantage of the s.c. route with respect to tolerability.

In conclusion, the results of the present study support the use of Merional^® ^by s.c. injection, that allows to obtain a similar effectiveness in terms of oocyte yield, a comparable incidence of adverse effects and cycle cancellation and probably has a better subjective tolerability with respect to the classical i.m. route.

## Competing interests

The author(s) declare that they have no competing interests.

## Authors' contributions

AC and AR drafted the manuscript and coordinated data collection in two of the Centers involved in the study. PA and LF coordinated data collection in the other two Centers involved. AR and IS collected data and provided statistical analysis, MM, NR and GDP participated in the design of the study, contributed to data analysis and manuscript preparation. All authors read and approved the final manuscript.
